# Gelam (*Melaleuca* spp.) Honey-Based Hydrogel as Burn Wound Dressing

**DOI:** 10.1155/2012/843025

**Published:** 2011-09-19

**Authors:** Rozaini Mohd Zohdi, Zuki Abu Bakar Zakaria, Norimah Yusof, Noordin Mohamed Mustapha, Muhammad Nazrul Hakim Abdullah

**Affiliations:** ^1^Department of Life Sciences, Faculty of Pharmacy, Universiti Teknologi MARA, 42300 Puncak Alam, Selangor, Malaysia; ^2^Department of Veterinary Preclinical Sciences, Faculty of Veterinary Medicine, Universiti Putra Malaysia, 43400 Serdang, Selangor, Malaysia; ^3^Division of Agrotechnology and Biosciences, Malaysian Nuclear Agency, Bangi, 43000 Kajang, Selangor, Malaysia; ^4^Department of Veterinary Microbiology and Pathology, Faculty of Veterinary Medicine, Universiti Putra Malaysia, 43400 Serdang, Selangor, Malaysia; ^5^Department of Biomedical Sciences, Faculty of Medicine and Health Sciences, Universiti Putra Malaysia, 43400 Serdang, Selangor, Malaysia

## Abstract

A novel cross-linked honey hydrogel dressing was developed by incorporating Malaysian honey into hydrogel dressing formulation, cross-linked and sterilized using electron beam irradiation (25 kGy). In this study, the physical properties of the prepared honey hydrogel and its wound healing efficacy on deep partial thickness burn wounds in rats were assessed. Skin samples were taken at 7, 14, 21, and 28 days after burn for histopathological and molecular evaluations. Application of honey hydrogel dressings significantly enhanced (*P* < 0.05) wound closure and accelerated the rate of re-epithelialization as compared to control hydrogel and OpSite film dressing. A significant decrease in inflammatory response was observed in honey hydrogel treated wounds as early as 7 days after burn (*P* < 0.05). Semiquantitative analysis using RT-PCR revealed that treatment with honey hydrogel significantly (*P* < 0.05) suppressed the expression of proinflammatory cytokines (IL-1*α*, IL-1*β*, and IL-6). The present study substantiates the potential efficacy of honey hydrogel dressings in accelerating burn wound healing.

## 1. Introduction

Honey is carbohydrate-rich syrup prepared by honey bees, derived from floral nectars and other plant secretions [1]. It is one of the most enduring materials to be used in wound care, attributed to its antibacterial, anti-inflammatory, and antioxidant properties [2]. Antibacterial activity of honey is due to its high osmolarity, pH, hydrogen peroxide production, and presence of other phytochemical components that originate from the nectar of plant [3]. There are numerous reports on the antibacterial activities of honey against a wide range of microorganisms resulting with acceleration of wound healing process [3–5]. The phytochemical component of honey is also responsible for its antioxidant activity which protects cells from the damage caused by free radicals thus decreasing the inflammation process [6]. It was reported that the free radical scavenging activities of honey is mainly due to the contents of flavonoids and phenolic acids [7].

Malaysian honey also demonstrated high antibacterial activity [8, 9] and was proven effective to treat wounds [10, 11]. Aljadi and Kamaruddin [12] reported that Gelam honey has antioxidative and radical scavenging properties, which are mainly due to its phenolic content. The extracts of this honey also demonstrated inhibitory effects against the inflammatory mediators in animal model [13]. Furthermore, we have previously reported that topical application of Gelam honey was effective to treat burn wound in rats [14]. However, when topical application of honey was administered onto the wound, rapid clearance from the wound site occurred as the honey tend to flow out from the wound area, making it difficult to maintain the therapeutic concentration over prolonged periods of time. These findings prompted us to further investigate the benefits of honey when incorporated into a hydrogel system. 

Numerous authors have reported on hydrogel technologies providing products suitable for biomedical application especially in wound management [15–17]. Hydrogels, with the features of moist wound healing and good fluid absorbance as well as having high water retention capacity, appear to be optimal media to enhance wound healing [15]. Thus, much interest has been focused on developing hydrogel-based wound dressings from a wide range of biomaterials as well as biologically active substances including chitin, chitosan, alginate, and sea cucumber [18–21]. 

In the present study, the composite matrix derived from honey and hydrogel dressing was introduced as a novel cross-linked honey hydrogel dressing having reservoir capacities for the sustained delivery of honey, which were prepared by irradiation technique. The development of a sustained release system would substantially increase the utility of incorporated honey in tissue repair and effectively interact with the burn wounds and thus facilitate healing. The present study reports the physical properties and wound healing potential of the prepared honey hydrogel in the management of burn wounds.

## 2. Materials and Methods

### 2.1. Honey Sample

Local monofloral *Apis mellifera* honey from the floral source of *Melaleuca *spp. (Gelam trees) was purchased from the Department of Agriculture, Malaysia. The samples were irradiated with 25 kGy gamma irradiation using radioactive source Cobalt 60 (model JS 8900) with the dose rate of 2 kGy per hour for sterilization purposes at MINTec-Sinagama, Malaysian Nuclear Agency, Bangi. Dosimetry was performed with ceric/cerous sulphate solution using potentiometric method of analysis.

### 2.2. Honey Hydrogel Dressing

Polyvinyl pyrrolidone (PVP) with molecular weight of 650 kDa (Kollidon 90) and Polyethylene glycol (PEG) with molecular weight of 400 kDa were obtained from BASF, Ludwigshafen, Germany. Technical grade agar was supplied by Oxoid. Hydrogel mixture of 15% (w/v) PVP, 1% (w/v) protein-free agar solution, and 1% (v/v) (PEG) were added with 6% (v/v) honey [22]. Briefly, the aqueous solution of PVP was prepared by dissolving 150 g PVP in 600 mL distilled water, and the solution was left overnight at ambient temperature (25°C). Agar solution was prepared by dissolving and heating 10 g of agar in 330 mL distilled water until clear and the mixture being stirred continuously before adding 10 mL of PEG. The homogenous mixture was left in the ultrasonic waterbath for one hour at 37°C, and 60 mL of honey was then added when the solution temperature reached below 45°C. A control hydrogel was prepared similarly without further addition of honey. The mixture was poured into plastic molds (5 cm in diameter; 3-4 mm in thickness) and left to set at room temperature before covered with polyethylene sheet and individually packed. The gels were cross-linked as well as sterilized by electron beam at 25 kGy at Alutron Irradiation Facility, Malaysian Nuclear Agency, Bangi (Model EPS-3000, conveyer speed of 4.4 m/minute, beam current of 10 mA, and energy of 3 MeV). 

### 2.3. pH and Swelling Analysis of Honey Hydrogel

Acidity/alkalinity (pH) test was achieved from aqueous extracts of hydrogel. The pH value was measured using the Mettler Toledo pH meter (model 320). Experiments were conducted in six replicates.

Swelling studies were performed on honey hydrogels in normal saline (0.9% NaCl) solution at room temperature. The weight of each hydrogel (*n* = 6) was recorded (Wd) prior to immersion in normal saline. The hydrogels were withdrawn from the solutions at predetermined time intervals, and their weights were determined (Ws) after first blotting with tissue paper to remove excess water. The swelling ratio (*q*) was calculated by *q* = (Ws − Wd)/Wd. Each swelling experiment was done in triplicate, and the average value was taken as the percentage of water absorption.

### 2.4. Animals and Experimental Designs

A total of 96 male Sprague-Dawley rats (weight 200–300 g) were used in this study and randomly divided into four experimental groups of 6 rats each. The experimental protocol was approved by the Animal Care and Use Committee (ACUC) of Faculty of Veterinary Medicine, Universiti Putra Malaysia (UPM) (reference no.: 08R36/July 08-Jun09). The animals were acclimatized to the laboratory conditions for one week prior to the onset of the experiment. The rats were individually caged and given commercial pellet and water *ad libitum* throughout the study. Rats were euthanized at 7, 14, 21, and 28 days after burn by an overdose of halothane inhalation, and skin samples were taken for histopathological analysis or snap frozen in liquid nitrogen and further maintained at −80°C until RNA isolation was performed.

### 2.5. Skin Preparation and Burn Lesion

Rats were anesthetized with an intramuscular (IM) injection of ketamine (50 mg/kg) and xylazine (5 mg/kg). Under anesthesia, the back and flank of both sides of the body were shaved. Following this procedure, rats were returned to their cages for 24 hours to allow any edema caused by the shaving procedure to recede. The experiment was conducted in accordance with the method reported in our previous papers [18, 22, 23]. Briefly, cylindrical aluminum templates (2.5 cm diameter × 3 cm length, a handle measuring 24 cm, and total weight 400 g) were heated in a water bath at a constant temperature of 85°C for 3 hours prior to inflicting burn areas on the skin of the rats. Five templates were heated simultaneously and used alternately and then were returned to the water bath to ensure the maintenance of the desired temperature of the template surface. 

Approximately 5 minutes elapsed between each use of a template. Rats were again anesthetized with an IM injection of ketamine (50 mg/kg) and xylazine (5 mg/kg). The anesthetized rat was positioned on sternal recumbency, restrained, and stretched on a metal stage. The location of the burn was marked between the last ribs and the horizontal line of the sacroiliac joints. Deep partial thickness burn was inflicted on the dorsal part of the rat between the last thoracic vertebra and the first sacrum by placing the heated and moistened template at right angles perpendicular to the dorsum of the rat on the premarked location for 5 seconds using an analogue stopwatch. Minimal and constant pressure was applied to ensure a perfect contact between the template surface and the skin. The shaved skin was smooth to ensure sufficient contact and uniform pressure over the entire lesion.

### 2.6. Treatment Protocol

All wounds in the treatment groups were dressed with either control hydrogel or honey hydrogels followed by Opsite film dressing (Smith and Nephew, Hull, England) as secondary dressing. The dressings were held in place with sterile gauze by suturing the gauze on rat's skin with coated vicryl 4/0 (Ethicon, Johnson&Johnson, Belgium) in a simple interrupted fashion. The covering of gauze gave mechanical protection of the dressings while Opsite film dressing was used to prevent hydrogels from being absorbed by the gauze thus permitting full utilization of wound healing properties of honey hydrogel. Sterile technique was utilized when changing the dressings every 7 days to minimize introduction of pathogens to the wound site. 

The hydrogels were easily applied to the wound with proper adherence and can be peeled off easily from the wound when the dressing needs changing without irritating the wound surface. Change of dressings was done every 7 days based on a pilot study done on the release profile of honey. The release of honey from hydrogel matrix displayed a sustained release profile up to 7 days. Opsite film dressing, a commercially available wound dressing preparation, was selected as the positive control group for comparison. The use of Opsite as a positive control was imperative in this study to ascertain that healing effects of the tested Honey Hydrogel dressing were due to the composition of the hydrogels and not as a result of secondary dressings. The negative control group received identical burn and environmental exposure, but no further treatment was given. 

### 2.7. Wound Area Assessment

All wounds were digitally photographed in the presence of a standard reference ruler. The wounds area was measured immediately by placing a transparent tracing paper over the wound and tracing it out. The tracing paper was placed on a 1 mm^2^ graph sheet and traced out. The squares were counted and the area recorded. The wound size measurements taken at the time of burn infliction and at the time of healing evaluation were used to calculate the percentage reduction in wound size, using the following equation:

(1) $$\text{Wound}\;\text{size}\;\text{reduction}\;\left(\%\right)=\frac{\left(A_{0}-A_{t}\right)}{A_{0}}\times 100,$$

where *A*_0_ is the initial wound area, and *A*_*t*_ is the area of the wound at predetermined time points (7, 14, 21, and 28 after burn day). The wound area was assessed by the same blinded observer.

### 2.8. Histopathological Analysis

Skin samples were fixed in 10% formalin solution and embedded in paraffin. Tissue sections of 4-5 *μ*m thickness were cut, stained with hematoxylin and eosin (H&E), and examined under light microscope. Digital photomicrographs were captured at representative locations using an image analyzer (Analysis LS Research) attached to a light microscope (Olympus BX51, Japan). The wounds were evaluated for the extent of reepithelialization, granulation tissue formation, architecture, cellularity, and inflammation.

### 2.9. RNA Extraction and Reverse Transcription Polymerase Chain Reaction (RT-PCR)

The midpoints of the wounds were bisected, snap frozen in liquid nitrogen, and stored at −80°C until analysis. Total RNA was extracted from wound tissues using RNeasy Fibrous Tissue Mini Kit (QIAGEN, Hilden, Germany) according to the manufacturer's instructions. Single-stranded cDNA was synthesized from the total RNA using MMLV reverse transcriptase (Promega, Madison, WI) and oligo (dT)_15_ as primer. Polymerase chain reaction (PCR) was performed using cDNA product with the sequence-specific primer pairs listed in Table 1 [24]. The housekeeping *β*-actin gene was used as reference. The thermal cycling condition set consisted of denaturation for 30 s at 95°C, annealing for 30 s at 60°C, and elongation for 45 s at 45°C with a final extension for 7 min at 72°C. A total of 30 PCR amplification cycles were performed using a Mastercycler thermal cycler machine (Eppendorf, Germany). The PCR products were electrophoresed through a 1% agarose gel and visualized by 0.5 *μ*g/mL ethidium bromide staining and UV transillumination using GelDocTM 2000 (Bio-Rad, USA). The band intensities were measured and quantitated by image analysis by comparing PCR products with *β*-actin.

### 2.10. Statistical Analysis

Data were expressed as means and standard error (S.E). Collected data were analyzed using the two-way blocked time effects ANOVA to determine the effects of treatment and time. The analyses were performed using the SPSS statistical package (SPSS, Version 15.0, Chicago, ILL, USA). *P* values of less than 0.05 were considered to be significant.

## 3. Results and Discussions

The prepared honey hydrogel and control hydrogel represent uniform, transparent sheets of three-dimensional networks with a thickness of 3-4 mm. They exhibited good transparency to allow monitoring of the healing progression as well as ensuring timely dressing changes. Honey hydrogel demonstrated a golden yellow discolouration due to the original colour of the honey itself which is yellow. 

The pH value of each hydrogel is shown in Table 2. Honey hydrogel was slightly acidic (pH 4.3) compared to control hydrogel (pH 5.3). This could be attributed to the acidic characteristic of honey itself with its pH being between 3.2 and 4.5 [2]. According to [25], dressings with a slightly acidic pH or similar to that of healthy skin (pH of 5.5) are most comfortable to wear, and the low pH creates an unfavorable environment for bacterial growth [26]. Furthermore, the acidic environment enhanced a maximum release of oxygen to meet the needs of the body's tissue repair [27]. 

**Figure 1:**
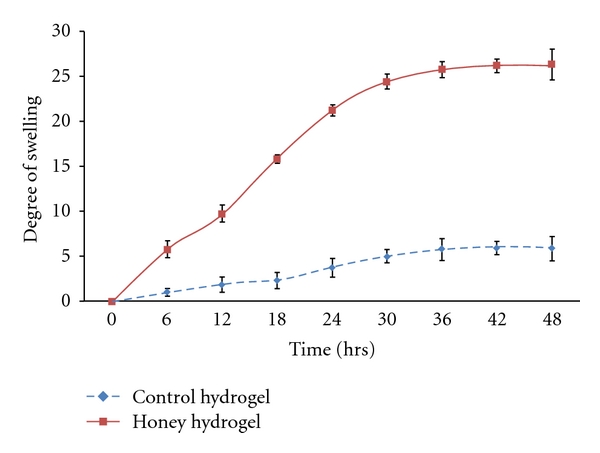
Swelling rate of honey hydrogels after immersion in normal saline at different time points (mean ± S.D). Honey hydrogel exhibited high capabilities in absorbing fluid compared to control hydrogel as it swelled significantly faster (*P* < 0.05) compared to control hydrogel at all time points.

The swelling ratio of the polymer was affected greatly by the incorporation of honey into the hydrogel system. As shown in Figure 1, honey hydrogel exhibited high capabilities in absorbing fluid compared to control hydrogel as it swelled significantly faster (*P* < 0.05) compared to control hydrogel at all time points. It could be surmised that incorporation of honey into the hydrogel system could beneficially affect the water absorption capacity of the polymer. This enhanced property could be attributed to honey high osmolarity created by its high sugar content. This result reveals that honey hydrogel could have the potential to prevent wound from accumulation of fluid by the absorption of exudates. Furthermore, hydrogels with high absorbing capabilities are recommended for exudative wounds such as burns [28]. 

**Figure 2:**
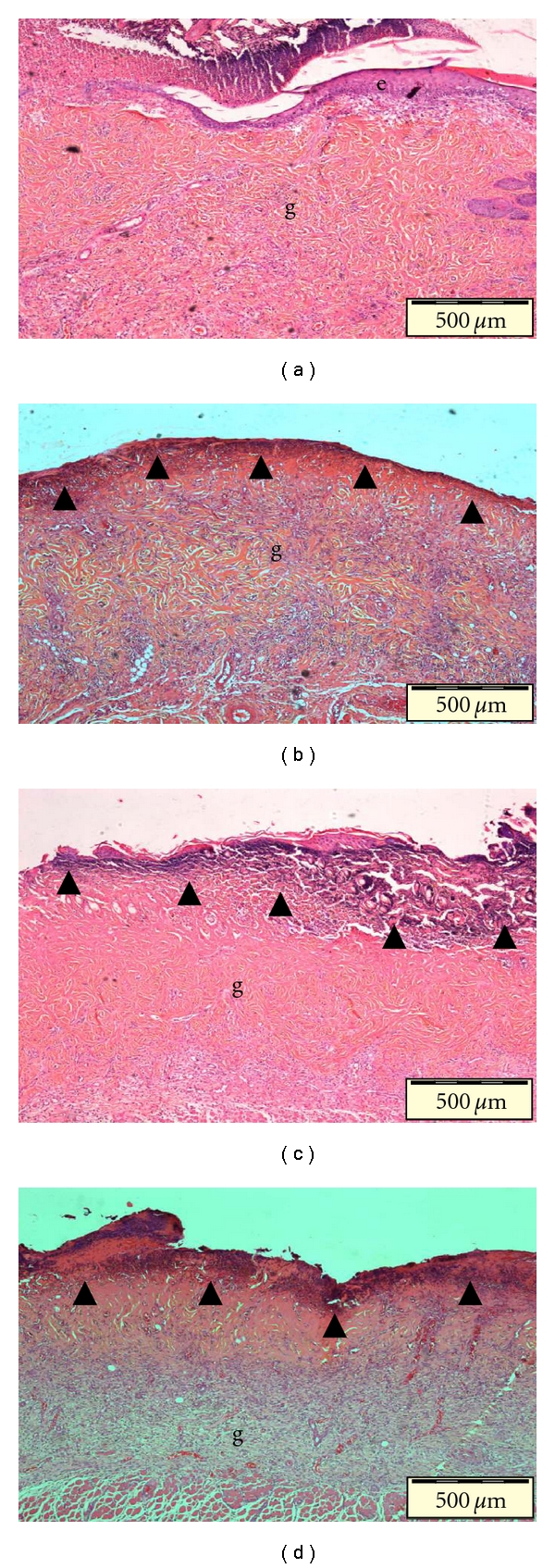
Representative photomicrographs of tissue sections at day 7 after burn stained with H&E. Early reepithelialization was seen in wound treated with (a) honey hydrogel. Homogenization necrosis of superficial layer of the dermis accompanied with inflammation response was observed in (b) control hydrogel; (c) Opsite film dressing; (d) untreated control wound. (e: epidermis, g: granulation tissue, arrowhead: necrosis; 40x mag.)

Honey hydrogel dressing significantly stimulated the rate of burn wound healing as demonstrated by increased reduction in wound size especially after 21 and 28 days after burn (*P* < 0.05) (Table 3). Microscopic evaluation demonstrated that there was a significant acceleration of dermal repair and advanced reepithelialization in wounds treated with honey hydrogel dressings as compared to other experimental groups (Figure 2). Histopathological comparisons showed that on day 7, honey hydrogel significantly attenuated inflammatory response (*P* < 0.05) as opposed to other dressings. Wounds treated with Honey Hydrogel also showed advanced granulation tissue formation, capillary formation and collagen synthesis. The early formation of granulation tissues seen in honey hydrogel-treated wounds might be due to the hydrogen peroxide generated by honey. Honey gives a “slow-release delivery” of innocuous concentrations of hydrogen peroxide, a substance which at low levels can stimulate angiogenesis and stimulate the growth of fibroblasts [2]. The increased rate of healing could also be attributed to the osmotic action of honey drawing out lymph, and thus providing a constant flow of nutrients from the functioning capillaries deeper down [29]. 

**Figure 3:**
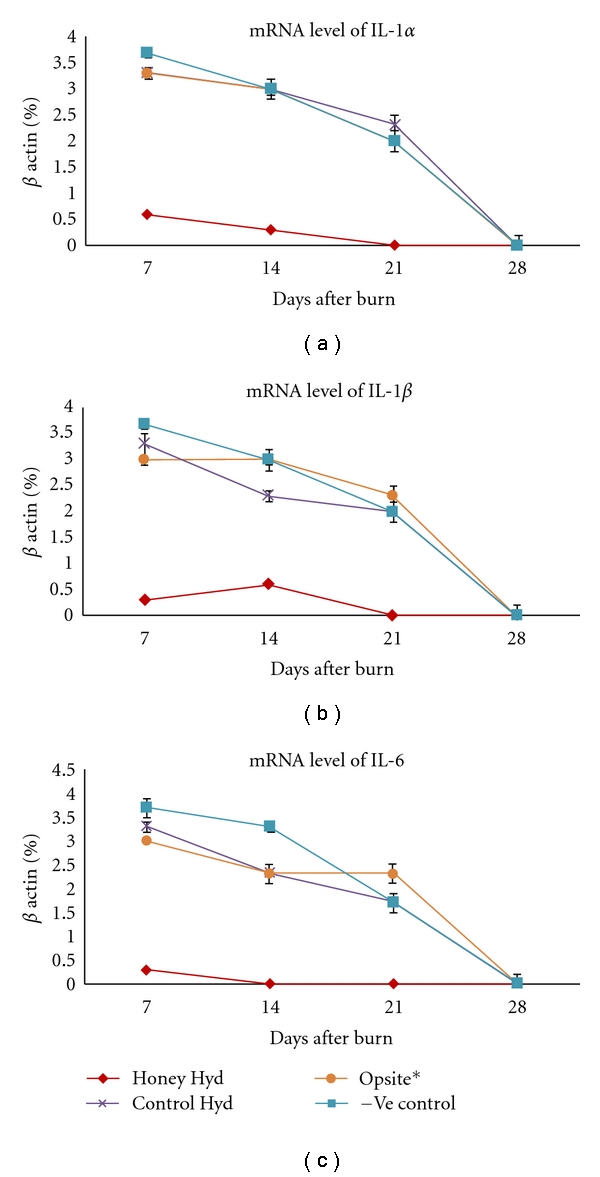
Modulation of cytokine profile by honey hydrogel: mRNA levels of (a) IL-1*α*, (b) IL-1*β*, and (c) IL-6 at various time points after burn injury, as determined by semiquantitative RT-PCR showing that honey hydrogel attenuates the proinflammatory cytokines.

At molecular level, this study also showed that Honey Hydrogel modulated the proinflammatory cytokines involved in wound healing (Figure 3). Burn wound is associated with a large amount of inflammation, which can lead to worsening of the tissue damage caused by the initial thermal injury [29]. Furthermore, it has been reported that excessive levels of proinflammatory cytokines within burn wounds impair the wound healing process [30]. This lack of amplification of the inflammatory cytokine cascade may be important in providing a permissive environment for wound repair to proceed [31]. Honey has been reported to reduce inflammation when applied to wounds, and its wound healing properties are mainly attributed to its antibacterial activity, anti-inflammatory actions, and antioxidant properties [2]. This study has clearly demonstrated that honey hydrogel has different effects on the various cellular elements and cytokines involved in the healing process. It is likely that accelerated wound closure in our burn model is attributed in part due to honey being released from the hydrogel matrix that acts in synergy with the moist environment provided by the hydrogel system.

## 4. Conclusions

In conclusion, honey hydrogel represents a feasible and productive approach to support dermal wound healing by regulation of multiple events that are central to the process of healing. The results on the physical properties of Honey hydrogel evidently showed that incorporation of honey into the hydrogel systems has altered the hydrogel matrix. Honey hydrogel dressings showed excellent physical properties such as having good transparency and the ability to absorb exudates, as well as demonstrating acidic pH value which met some of the essential requirements of an ideal burn wound dressing. The resultant wound healing effects were attributed to the synergistic effect of the hydrogel matrix and the incorporated honey. These treatment outcomes make our dressing highly promising as an alternate wound care system for the treatment of wounds and certainly opening new avenues for future research and development.

## Figures and Tables

**Table 1:** Sequences of primers and the size of products produced based on previously published work [24].

Primer	Direction	Sequences (5′ to 3′)	Product size (bp)
*β*-Actin	Forward	GTG GGC CGC TCT AGG CAC CAA	540
	Reverse	CTT TAG CAC GCA CTG TAG TTT CTC	

IL-1*α*	Forward	ATG GCC AAA GTT CCT GAC TTG TTT	625
	Reverse	C TGG TCG GGC ACA ACG ACT TCC	

IL-1*β*	Forward	ATG GCA ACT GTT CCT GAA CTC ACC T	563
	Reverse	TT TCC TTT CTT AGA TAT GGA CAG GAC	

IL-6	Forward	ATG AAG TTC CTC TCT GCA AGA GAC T	638
	Reverse	CTC TAG ATG AGC CGT TTG GAT CAC	

**Table 2:** The pH value of honey hydrogel and control hydrogel. Results are expressed as the mean of 6 replicates ±SD.

Sample	pH value
Honey hydrogel	4.34 ± 0.02
Control hydrogel	5.26 ± 0.02

**Table 3:** Measurement of wound size at 7, 14, 21, and 28 days after burn presented as percentage of wound size reduction.

Groups	Percentage of wound size reduction (%) (mean ± SD)			
	Day 7	Day 14	Day 21	Day 28
Honey Hyd	15.42 ± 2.1^b^	41.11 ± 3.4^b^	81.78 ± 0.9^c^	91.27 ± 0.3^b^
Control Hyd	13.44 ± 1.5^ab^	40.35 ± 4.3^b^	58.94 ± 2.1^a^	72.75 ± 1.8^a^
OpSite	12.27 ± 1.7^ab^	39.63 ± 2.1^b^	60.06 ± 1.3^a^	78.54 ± 1.0^a^
−ve Control	10.80 ± 1.2^ab^	28.01 ± 1.9^a^	56.72 ± 0.7^a^	70.86 ± 2.9^a^

^a,b,c^Means with different superscripts within a column were significantly different at *P* < 0.05.
